# Amputation of the Femur

**Published:** 1879-05

**Authors:** 


### Miscellaneous.

### Surgical Notes from Berlin.

[From the Chicago Medical Journal and Examiner.]

Amputation of the Femur.—A man aged 36 years, suffering from a malignant disease of the lower end of the femur, was brought into the amphitheater, and while the assistants were administering chloroform to the patient, and making other necessary arrangements for the amputation, Prof. Langenbeck made the following general remarks on the subject:

"Where it can be done, I make *side* flaps, instead of antero-posterior ones, as is generally done. I do this, first, because a better drainage can be effected after the operation, and second, because I believe any resulting cicatrix is more likely to be situated at a point that will render it less liable to be irritated by the friction connected with the movements of the stump.

"Again, I make my flaps *entirely*, of skin and its immediately adjoining tissues. I want no muscular tissue overlapping the end of the bone. If you have a long muscular flap, it is sure to either slough away or gradually become absorbed. If it should slough, it immediately complicates your case by adding to the dangers from septicæmia."

When he had made his skin flaps he then made a section of the periosteum, extending half way around the shaft of the bone, and six centimeters in length. Having peeled this carefully back, he had a perfect covering for the end of the bone, after the latter was sawn off. He says, that since he has adopted this mode of procedure, he has never had a single case of secondary disease, such as caries or necrosis, following his amputations. When he had sawn off the femur, and brought the skin over the end of the stump, lo! his flaps were so short that they would not come together without considerable tension. He remarked: "As you see, gentlemen, the end of my bone is either too long, or my flaps are too short, and, as I cannot lengthen the latter, I shall have to shorten the former;" and without another word of explanation he proceeded to saw off another six centimeters from the end of his stump.

Before he closed the wound, he applied eighteen ligatures, all of which, except the one to the main branch of the femoral artery, were of carbolized catgut. These ligatures of course were all cut short and left to be absorbed; and I have never seen him leave the long ends of ligatures out of a wound with a view to their future removal. A drainage tube was introduced into the inferior and superior border of the stump, and the whole was sewed together in the most painstaking manner. The stump was dressed with Lister's antiseptic dressing, the whole operation being done under the spray. The operation lasted one hour and ten minutes. In contrast with this time, I have seen Prof. James R. Wood, of New York, do this operation complete in five minutes, making his flaps and sawing off the bone in forty-five seconds.

The result of this operation was most satisfactory indeed. The whole wound healed by first intention, and the patient never had a temperature above 37.5, and never suffered any considerable amount of pain.

In speaking of the rapid recovery of this case, Prof. Langenbeck said that he attributed it mainly to the painstaking manner in which he had arrested *every vestige* of hæmorrhage before closing the wound. He believes that in the vast majority of cases where a constitutional reaction followed surgical operations of this kind, and which we had formerly been taught to look upon as having its origin in an inflammatory reaction, was in fact of a septic character, and that a decomposed blood clot situated at the extremities of open blood vessels, was the very best possible condition for generating such a process.

